# Adaptation and content validation of measure yourself medical outcomes profile (MYMOP) for 7–11 year-old children

**DOI:** 10.1007/s11136-024-03702-3

**Published:** 2024-06-14

**Authors:** S. Ishaque, R. M. Roberts, J. Karnon, D. Thomas, A. B. Salter

**Affiliations:** 1 School of Public Health, The University of Adelaide, Adelaide, South Australia Australia; 2 College of Medicine and Public Health, Flinders University, Adelaide, South Australia Australia; 3 School of Psychology, The University of Adelaide, Adelaide, South Australia Australia; 4 Women’s and Children’s Hospital, Adelaide, South Australia Australia

**Keywords:** Health related quality of life, Individualised, MYMOP, Paediatric MYMOP, Patient centred outcomes

## Abstract

**Background:**

The Measure Yourself Medical Outcome Profile (MYMOP) is an individualised tool designed for adults but used with children without any evidence of validation in this population. Individualised instruments are patient-specific rather than disease-specific and therefore can be applied across various health conditions. This study sought to adapt, and content validate the MYMOP for application in 7–11 year old children.

**Methods:**

There were two main phases of the four iterations: expert consultation (three rounds) and interviews with child-parent pairs at the Outpatient clinics of a Children’s Hospital. Thematic analysis was undertaken using an inductive, interpretative approach.

**Results:**

Four paediatricians completed the first survey, five paediatricians participated in the focus group, and four paediatric health-related quality of life (HRQOL) research experts completed the second survey. Several changes were recommended to the MYMOP by the expert groups. Twenty-five children (17 general medicine, and 8 diabetes/endocrine clinic) aged 7–11 years completed the draft paediatric MYMOP (P-MYMOP) and were interviewed. Results demonstrated that the majority of participants were able to identify their own problems and activity limitations, and all participants understood the 7-point faces scale. Most parents and children perceived that the P-MYMOP would be useful to complete before clinic appointments.

**Conclusions:**

The P-MYMOP is the first content-validated generic individualised HRQOL measure for children 7–11 years old. Given that validation is an iterative process, further research to assess its feasibility, reliability, and construct validity is required.

## Plain English summary

This study was conducted to change a health-related quality-of-life (HRQOL) measurement tool, Measure Yourself Medical Outcome Profile, to make it appropriate for use in children 7–11 years of age. This study produces the first ever individualised HRQOL tool for use in children, which is likely to support inclusion of their voices in their clinical care. The adaptation/change was conducted by a multi-step process that involved consultation with paediatricians, research experts, children, and their parents/caregivers. The multi-step process ensures that all stakeholders find the new tool helpful and appropriate. Also, inclusion of stakeholders is likely to support the use of this tool (Paediatric Measure Yourself Medical Outcome Profile) in future during routine clinical care of children.

## Introduction

Individualised Health-Related Quality of Life (HRQOL) measures are tools that lack pre-determined domains. These tools provide respondents the opportunity to select their concerns that are important to them at the time of tool completion. Individualised measures are superior in identifying patient needs, values, and goals [1, 2]. However, like any other measurement approach [3, 4], these tools have some limitations, and cannot be used for between individual/group comparisons or economic evaluation [1, 2].

At present, validated, individualised HRQOL measures are available for use in adults [5–9]. However, individualised HRQOL tools for paediatric populations are non-existent. The Measure Yourself Medical Outcome (MYMOP) is a validated, adult, individualised measure that allows respondents to name and score two priority symptoms, an activity limited due to the selected symptoms and their overall wellbeing [10]. A literature review of the psychometric properties of the tool and its adaptation confirmed lack of validity for children [11]. The MYMOP was found suitable for validation in a paediatric population based on the personal experience of the *CARE Program for Integrative Health & Healing,* Department of Pediatrics, University of Alberta. The tool was used successfully in the *Program* given that it was simple to use and avoided non-applicable questions to developmentally delayed children.

This study was designed to adapt and validate the MYMOP for use in a paediatric population of children aged 7–11 years based on their established homogeneity in cognitive abilities [12–14].

A mixed-methods research design including interviews, focus group, and survey was used. This approach is considered appropriate for instrument adaptation [15, 16]. Methods and results of each iteration are described separately below.

### Sampling logic

To ensure relevancy in a clinical setting, the paediatric MYMOP (P-MYMOP) was adapted in consultation with multiple stakeholders.

**Fig. 1:**
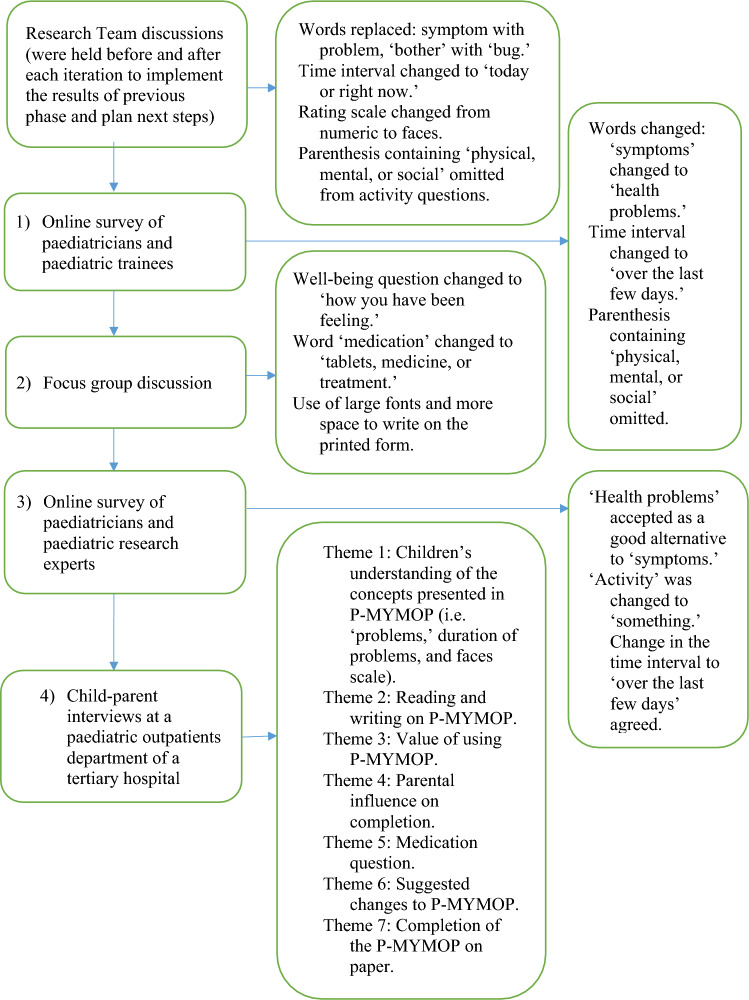
Iterations in the adaptation of the Paediatric MYMOP and their outcomes

There were four iterative stages of this study. First, a group of paediatricians was consulted via an online survey, followed by a focus group. Next, Australia-wide paediatricians and paediatric researchers from a quality-of-life special interest group were consulted to provide further refinements. This included academics and clinicians interested in the advance and effective use of patient reported outcomes measures in clinical settings. Lastly, children and their parents/guardians were consulted to suggest further changes to the content and test whether the P-MYMOP had appropriate content for children (Fig. 1). Participants were recruited until saturation was reached [17], which was defined as the emergence of no new information.

Ethics approval for the first and second iteration (HREC/16/WCHN/8) and fourth iteration (HREC/17/WCHN/36) was obtained from the Women’s and Children’s Health Network Human Research Ethics Committee, for the third iteration was obtained from the University of Adelaide’s Human Research Ethics Committee (H-2016–270).

## First iteration

### Methods

An online survey was distributed to 10 paediatricians through staff email addresses in the Department of General Medicine at the Women’s and Children’s Hospital (WCH), Adelaide.

**Fig. 2:**
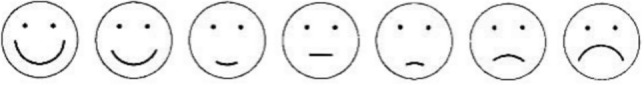
The seven-point faces scale of the Paediatric MYMOP

It included questions relating to suggested changes in wording and layout of the MYMOP (Fig. 1), and inclusion of a relatively simple set of seven faces [18] as a scale for children aged 7–11 years old (Fig. 2).

Quantitative data were analysed descriptively using basic counts, and qualitative responses were analysed with a summative approach to qualitative content analysis [19]. The principal researcher (SI) identified and quantified certain words in the participants’ text responses to explore usage and interpreted them via discussion with the research team. At the start of the study and after each iteration, decisions about the changes in the P-MYMOP were made by the research team to reflect the developmental stage of the target population and the feedback from participants.

### Results

There were four responses (4/10) to the survey. All respondents agreed to changing the word ‘symptoms’ to ‘problems;’ and the deletion of words like ‘physical, mental, or social’. Furthermore, substitution of the word ‘activity’ with ‘thing” was also suggested. After research team discussion, the term ‘problem’ was changed to ‘health problems’ and the term ‘thing’ changed to ‘something’ in the activity question. Three respondents did not agree with changing the word ‘bother’ to ‘bug,’ therefore it was retained.

There were three different responses to the selection of the appropriate time interval for the P-MYMOP: ‘over the last week, ‘over the past few days,’ and ‘today.’ A fourth respondent did not have a preference. After research team discussion, the time interval was changed to ‘over the last few days,’ with the decision to test this change in the next iteration.

There was a 50/50 split among respondents on the use of a ‘faces scale’ for P-MYMOP, which required further discussion in the next iteration.

The medication questions were considered appropriate for primary caregivers of children.

## Second iteration: focus group discussion with paediatricians

### Methods

An in-person focus group of approximately 20 min was conducted with paediatricians at the Department of General Medicine WCH to discuss the results of the first online survey, facilitate discussion concerning the use of P-MYMOP in hospital setting, and plan the next stages of validation.

The P-MYMOP draft Tool, prepared based on the first survey responses, was presented to the participants for discussion, along with results from the first iteration. Resulting data were analysed using the summative approach to qualitative content analysis [19].

### Results

Five paediatricians participated in the focus group. Based on the discussion and agreed up on decisions of the group, the well-being question was simplified to ‘how you have been feeling?’. The medication question was changed to ‘are you taking any therapy or treatment?’ and the word ‘medicine’ preferred over ‘medication.’ After research team discussion, the medication question was changed to ‘do you take any tablets, medicine, or treatment?’ and was open to further validation in the next iteration.

Further suggestions from the group included use of a large font and more spacing for responses. It was also suggested that the P-MYMOP be completed in a waiting room before a scheduled appointment with a paediatrician.

## Third iteration: online survey of paediatric research experts and paediatricians across Australia

### Methods

A second online survey was designed to consult with paediatric research experts and paediatricians across Australia. The survey included questions about the appropriateness of the changes made in the first two iterations.

The survey was circulated to the Australian members of the International Society of Quality of Life Research (ISOQOL)—Child Health Special Interest Group as a representative body of researchers interested in child HRQOL/QOL (n = 22), and to paediatricians via the monthly Newsletter of the Paediatric and Child Health Division of the Royal Australian College of Physicians. One reminder was sent to potential participants. Resulting data were analysed using similar methods reported in the first iteration.

### Results

Four responses were received from paediatric researchers. No additional responses to the reminder included in the Newsletter were received therefore the survey was closed.

Of the four respondents, three agreed to asking children to name their ‘health problems,’ confirmed the use of the time interval ‘over the last few days,’ and the replacement of the word ‘activity’ with ‘something’.

All respondents confirmed that the ‘faces scale’ would be beneficial to the paediatric tool. After discussion, the research team changed the term ‘health problems’ to ‘problems’ on the P-MYMOP, to widen the scope for young children.

## Fourth iteration: child-parent interviews at the women’s and children’s hospital’s paediatric outpatients department

### Methods

This iteration involved completion of the P-MYMOP and interview of children aged 7–11 years old and their parent/guardian visiting the Department of General Medicine or Diabetes/Endocrine Outpatient clinic at the WCH Adelaide. A child-parent/child-guardian pair was eligible to participate in the study if: (1) the child was 7–11 years old; (2) the parent/guardian and child could speak and read English; and (3) child was able to name their own issues. After informed consent was obtained, the parent/guardian was asked to complete a demographic information form followed by completion of the P-MYMOP with the child. A semi-structured interview was later conducted with the child and parent/guardian discussing the wording, layout, any perceived benefit of completing the tool and willingness to complete the P-MYMOP as a routine task before their clinic appointment. This iteration involved a mixed-methods approach given that demographic information, P-MYMOP responses, interviewer observations, and interview responses were collected. The session was audio recorded and later transcribed. Based on the diagnosis of children, the clinic nurse determined which children booked for appointment for that day would be able to complete the MYMOP by themselves and only those children-parent pairs were approached for recruitment. The children who had severe developmental issues and were non-verbal were not approached.

Analysis of the interview data was performed according to the Braun and Clarke guidelines on reflexive thematic analysis [18–21]. This study used an inductive, interpretative theoretical approach, adopting a critical realist research paradigm [21]. This paradigm (realist epistemology) means we were interested in understanding and reporting the participants own understandings and own meanings, without assuming an objective truth.

### Results

This phase of the study ran for 16 weeks and a total of 25 child-parent pairs participated. Data resulting from a pair with a non-verbal child unable to complete the tool himself, was excluded from analyses. Demographic characteristics of participants are presented in Table 1. The participating children’s ages were representative of the target population. Of the 24 children, 20 were native English speakers. Sixteen children were from the General Medicine Clinic and eight children from the Diabetes and Endocrine Outpatient clinic.

**Table 1:** Demographic Characteristics

No	Age	Child’s gender	Mother/father/guardian	1st language	Parent’s report of presenting complaint/possible diagnosis	Problem 1	Problem 2	Activity
GM1	7	M	Mother	English	Behavioural problems	Happy	Not sure	(Left blank)
GM2	10.5	M	Mother	English	Sleep apnoea	Not getting work done	People calling me names	Get 200 tasks done
GM3	8	M	Mother	English	Auditory processing disorder & dyslexia	Sore toe	Learning, auditory and sensory processing diagnosed 3 months;1 year ago had for at least 1–5 years	No
GM4	8	M	Mother	English	Obesity & behaviour issues	Being blamed for things he hasn’t done	When things are hard to do	Unable to finish school work
GM5	10.5	F	Mother	English	Enlarged liver, raised liver function, increased IgE level	Bullies at school	Bullies get rewarded for bad behaviour	Swimming because of my eczema
GM6	8	F	Mother	English	Follow-up	Not allowed to have too much lollies	When I fall down and scratch myself	I can’t play on the monkey bars
GM7	7	M	Mother	English	Asperger’s	My sister annoys me	I want to have a normal break at school	Playing on Xbox 360
GM8	8	F	Mother	English	Diagnosis of small height and weight	Noise issues	(Left blank)	Doesn’t stop me
GM9	8	F	Mother	English	Migraines	(Left blank)	(Left blank)	(Left blank)
GM10	10	M	Mother	English	Autism	Getting off the internet	Going to school and doing work	Is to find it easy to learn
GM12*	10	F	Mother	English	Cerebral Palsy	Cutting my hair	Go to sleep	School
GM12a*	11	M	Mother	English	Asthma	(Left blank)	(Left blank)	(Left blank)
GM13	7	M	Mother	English	(Left blank)	School	Stomach	Football
GM14	8	M	Mother	English	Mozaki Chromosomes	Fight	Mum hard at act	Stop getting angry
GM15	10	F	Mother	English	Seizure of unknown cause	My mum getting angry with me	Dying	Feeling happy
GM16	7	F	Mother	Malayalam	Follow up after neurosurgery	(Left blank)	(Left blank)	(Left blank)
Endo1	11	F	Mother	English	Growth delay	(Left blank)	(Left blank)	(Left blank)
Endo2	8	F	Mother	English (non-native)	(Left blank)	Scared	(Left blank)	Jumping into the water
DM1	10	M	Mother	Arabic	Type 1 diabetes	Coming here and missing school	Staying away from my family for a long time	Make a new kind of pump
DM2	7	M	Father	Punjabi	Type 1 diabetes	Insulin injections	Reading/studies	Can’t eat sweet stuff like ice cream
DM3	8	F	Foster mother	English	Type 1 diabetes	Food	Mood	Making friends
DM4	8	F	Mother	English	Type 1 diabetes	Getting interrupted in school	Always having to check by readings	(Left blank)
DM5	7	F	Father	English	Type 1 diabetes	Erin (older sister) has got to eat sweets	Sweets	Eat sweets
DM6	8	F	Mother	English	Type 1 diabetes	About my friends arguing at school	One of my friends does not like to play with my other friends	Make a friendship club

^*^GM12 and GM12a were siblings

The completion of the P-MYMOP was followed by a semi-structured Interview. Children were also observed during their completion of the P-MYMOP to determine whether they understood the concepts presented in the tool. Six themes were generated from the coded data collected:

1. children’s understanding of the concepts presented in the P-MYMOP (i.e. ‘problems,’ duration of problems, and ‘faces scale’)
2. reading and writing on the P-MYMOP
3. perceived value of using the P-MYMOP
4. approach to the medication questions
5. suggested changes to P-MYMOP, and
6. connecting activity with identified problems.

Results for these themes are reported below and their supporting codes from the data are presented in Table 2.

**Table 2:** Themes and codes

Themes	Themes & subthemes defined	Subthemes	Codes
1) Children’s understanding of concepts presented in P-MYMOP (i.e. ‘Problems,’ ‘Duration of problems,’ and ‘Faces scale’)	If children can name their problems, they can complete individualised questionnaires Child’s identification of his/her problems tells us that they can understand the P-MYMOP and hence provide positive mark to the content validity of our questionnaire	1. Child named their own problem	C: (thinking…and then wrote down something on the form -observation) is that it?’ M: What do you think, what your problem would be? C: Hmmmm you can write… M: You can write it down. Come on (handed the pen to child) Observation: Child is completing questionnaire on her own. Getting help with spellings M: (helping with spelling) u i n g Example when parent named the problem for child: GM3: Mother: to do you want to fill out this? This is like a visual way for us instead of using words So, you can write up here. You can say sore toe Mother: No no no, you just write on the smiley faces. You show me, show the lady how good or how bad it is you finding learning, reading, and writing at the moment
		1. Children’s understanding of the faces scale	DM3: M: Now look at the faces below and circle the face that shows how bad that problem has been over the last few days C: Hmmmm M: So how bad it has been over the last few days. Have you thought about food lots, not a lot, or (pointing to faces) C: Hmmm what do you think? M: No this is your question C: Hmm I think this one DM5: F: now, look at the faces below and circle the face that shows how you have been feeling over the last few days.’ Observation: child selects a face
		1. Understanding of the duration of problem	DM2 F: How long have you had problem 1. This problem (pointing to questionnaire) C: Since a year F: 3 months to 1 year DM3:C: How long have you had problem 1 either all the time or on and off please circle (reading the duration question) C: I have had it for five years M: How long have you had problem with food? C: Five years M: Over five years C:No five years’
2) Reading and writing on P-MYMOP	This theme included observational data if the participating children were able to read and write on the P-MYMOP	1) Who wrote the individualised items on P-MYMOP	Example of getting help with spelling: GM3: Child: how do I spell sore? Mother: You tell me how you spell sore? Child: s o r? Mother: Hmmm (affirming) and e.hmm DM5: F: You can tell daddy if you want him to write C: I want to write
		2) Who read P-MYMOP	DM5: Codes from interview of a 7 year old child from diabetes clinic: Interviewer: Could you understand the questions when you were reading them or when your dad was reading? C: Indicated no (observation) I: No? Was it confusing? C: No I just can’t read but I know what daddy said
		3) Circle/cross to Select a face on the paper form	Participants completed paper forms were evaluated for this subtheme. Details are given in the results section
3) Perceived value of using P-MYMOP		Value of using P-MYMOP	GM15:’ Mother: yeah for sure. Yes absolutely which I guess for children often parent is the one who does talking so I guess this is an easy way to get them to be able to comment without having to may be face to face with the doctor GM3: So this questionnaire, and I really like the visuals (referring to faces scale) because that is something they really can use and often in learning you use visuals in every element of learning for kids, specially kids with developmental and learning difficulties that you rely on visual stimulus a lot and that’s really handy It’s a great scale and a great questionnaire for GP but for specialists … a specialist or a paediatricians for longer term care they might not necessarily understand that there may be even an issue and therefore a questionnaire for parent might be a better thing. As I said when we were at (hospital) with his broken arm I loved the fact they talked to him about his body (referring to his son) and as a parent often I find that very refreshing And they hate, kids hate being talked about. [Name] coz she really have had a life time of medical appointments about her. She really really hates being talked about in her presence and I often, if I can get to see the therapist by making appointment to see them (Interviewer: Before?) before to meet with them to discuss it so that I don’t have to say it in front of her GM3: Still a good scale to use, I do love … I love the use of visuals. Any part of you know teaching kids or communicating with them they work really well GM5: M: Also it is a very good idea, isn’t it? (asking child) It’s about the emotional side of how they are feeling as well as physical. I: So do you see value in that? M: oH yeah. Yeah I do a lot for sure
		Interested in completing in future	GM14: M: I reckon, it should be done. Because like sometimes you forget what you forget stuff that you wanted to ask and if the kids can it themselves it will make it a lot easier for all of us
		Statements on child’s voice in consultation	DM3: M: Well it is (P-MYMOP) beneficial. We actually see someone else outside of here for these problems so for the doctors to know the problems that are going on. … I think would help M: Do you like this? Being able to give this to Dr (name) or would you rather tell Dr (name)? C: Give it to Dr (name) M: right. Give it,give something like that to her C: so she knows what I am saying C: Coz it will be difficult to explain it to her M: Yeah … C: …very difficult ‘
4) Approach to the meditation questions	Completion of medication questions on the paper form and any issues raised about the medication questions in the interview		DM1: M: (medication) with this one I believe it wasn’t much relevant to the insulin because they are all type 1(diabetes). In their case it’s all type 1 All attending the hospital are type 1 and they are taking insulin so it was a bit confusing DM2: ‘: ok this is about the medications if he is taking anything. He is taking insulin obviously but is taking anything else? F: No I: OK then just leave this out F: It’s not applicable Dm5: F: Yes unfortunately we cannot cut insulin DM6: M: Is avoiding it … It’s quite important that she takes it. …. Not important
5) Suggested changes to P-MYMOP			As observed by the researcher from the P-MYMOP forms, the children from the Diabetes/Endocrine and General Medicine Clinic whose parents suggested changes, did not have any presenting complaints DM1: M: yeah. If you can please give us more space to write down that we are here to see the educator, the doctor and seek guidance and speak to this and this. Aaah … couple of issues DM2: F: I have done this thing in my own masters. I have done an MBA so I have done this research program and sort of thing If you … If you make this questionnaire related, more specific towards diabetes. For example, are you … are you happy? First of all you need to tell this kind of faces, how happy you are with your diabetes … You will you will understand from kids point of view whether kid is happy with diabetes or not, one thing (observation: child pointed to a face) F: See this is a specific answer. I didn’t tell him anything. This is first question. Second question is OK the way you are taking insulin, is that insulin is helping you control your Hbgl levels. So what other help you need in terms of controlling it Endo1: M: I guess. With it says ‘problem’, it’s kind of like, I don’t know. This doesn’t really make sense. Yeah I: What would make sense? Can you suggest a different word? M: I don’t know. Any worries or concerns I guess. May be. I don’t know GM6: M: (reading the activity question to point out the place of confusion for her) Look at the faces below and circle the face that shows how hard it has been to you. I think there is something wrong with the instructions there coz I got confused How hard it has been to you. … I think has been for you, for you not to be able to do this for the last few days?
6) Connecting activity with identified problems	The theme is about the completion of the activity question on the P-MYMOP, and any issues raised in the interview about the question		DM 1: M: No, he separated the problems from what he really wants to do. So we believe that this question is separated, totally separated from the second question. When he expressed his feelings here about the problems he didn’t believe that this problem would stop him from (I: doing anything) doing anything DM6: M: OK choose something you really want to do but find it hard because of your problems. I think it’s about these problems right. So what did you want to do that is hard? C: Writes down something M: Hmm that is tricky M: But that doesn’t make you super sad like your other ones? C: No

#### Theme 1 Children’s understanding of the concepts presented in the P-MYMOP (i.e. ‘Problems,’ Duration of problems, and ‘Faces scale’)

Theme 1 had three sub-themes: (1) child named their problem; (2) the child’s understanding of the ‘faces scale’; and (3) understanding of the duration of the problem.

Information about a child’s ability to name his/her problem and their understanding of the ‘problem duration’ came from the interviewer’s observation during completion of the P-MYMOP by the child-parent pair. Data on understanding of the ‘faces scale’ was both observational and in response to the direct questions about the children’s impression of the ‘faces scale.’

Twelve children from the General Medicine (12/16) and six children from the Diabetes/Endocrine cohort (6/8) were able to name their problem and measure the effect of it using the ‘faces scale’. Six of the participating children were not able to identify their problems, in which case parents/guardians either provided them with some examples or reminded the children of recent discussions about the problem. With this help,12/18 participating children were able to name their problems.

Of the six children (4 General Medicine, 2 Diabetes/Endocrine) who did not name their problems, four were attending the Clinic for follow-up appointments and had no presenting complaints. In two cases (1 General Medicine, 1 Diabetes/Endocrine), parents chose the problems independent of the child; but proceeded to ask the child to score these on the ‘faces scale’.

The ‘faces scale’ was unanimously accepted as helpful by all participating children and parents. All the children understood the’problem duration’ question on the P-MYMOP.

#### Theme 2 Reading and writing on the P-MYMOP

There were three sub-themes under the main theme of ‘reading and writing on the.

P-MYMOP’: (1) who wrote the individualised items on P-MYMOP; (2) who read the P-MYMOP; and (3) completion of the ‘faces scale.’

Data about this theme was gathered through observing the child-parent pair during completion of the tool. No issues were identified with reading, and all participating children understood the tool, as it was permissible for either child or parent to read the tool. Similarly, writing individualised problems on the P-MYMOP form was not an issue. In some cases, the children needed help with spelling which was provided by their parents.

To explore the method of face selection to score the questions on the P-MYMOP, the participants’ completed paper forms were examined. Participants selected faces either by ticking, filling with ink, making a cross, underlining, or a combination of circling and ticking. These responses demonstrated that the children understood the instruction of selecting a face by making a mark on them and it appeared to be intuitive for them to do so.

#### Theme 3 Perceived value of using the P-MYMOP

There were two sub-themes under the main theme of ‘value of using the P-MYMOP’: (1) interested in completing in future (as routine practice) and (2) statements on the value of their child’s voice during consultation.

Of the 24 participants, 21 perceived the use of the P-MYMOP as beneficial for routine clinical practice. Parents/guardians believed that the P-MYMOP would help their child identify and remember important issues to discuss with their doctor and provide them with an opportunity to express themselves in a setting where they were less likely to do so.

Three parents did not see any value of using the P-MYMOP for their children. A common feature of these interviews was that the children involved were visiting the Clinic as a follow-up appointment and had no current complaints. Twenty-one of the study participants perceived the completion of the P-MYMOP as valuable and were willing to complete the tool in future before their clinic appointments as part of usual clinical practice.

#### Theme 4 Approach to the medication questions

The medication questions were completed by the parents of 12/16 General Medicine patients. All parents stated that their children were not taking any prescription medication, however two stated that their child had been taking over-the-counter and/or herbal remedies which they did not consider ‘medication.’ No parents from the General Medicine cohort considered the medication questions irrelevant.

In the Diabetes/Endocrine Clinic, one parent did not complete the medication section on the form. Of the five parents who completed the tool, one clearly mentioned that the section was not relevant for patients with diabetes, and the rest of the parents also had concerns, as is evident in the data codes.

Data suggest that medication questions are not relevant for children with diabetes, as the P-MYMOP inquires if patients would like to stop taking their medication—which is not possible for patients with Type 1 Diabetes. For patients visiting the General Medicine Clinic however, these questions can help capture information about alternative therapies/herbs/over-the-counter medicines.

#### Theme 5 Suggested changes to P-MYMOP

Three parents from the Diabetes/Endocrine Clinic, and one from the General Medicine Clinic suggested some changes to the P-MYMOP. Their corresponding children did not have any current complaints and therefore left the first two questions on the P-MYMOP blank. The changes suggested were more options for the number of problems, asking more disease-specific questions, and changing the word ‘problem’ to ‘worry’ or ‘concern.’

One parent from the Diabetes/Endocrine Clinic, whose child enthusiastically completed the questionnaire, and who also mentioned that P-MYMOP would be a good preparation tool prior to clinical consults, suggested more writing space for problem descriptions. Another parent from the Diabetes/Endocrine Clinic did not like the P-MYMOP and found it too generic, suggesting alternative focused questions.

Inspection of the eight self-completed questionnaires showed that the spaces provided for children to write were not large enough to allow for the size of their handwriting. The written responses to problems/activity questions were long and did not fit easily in the space provided. Therefore, a decision was made to provide increased space for three individualised questions. Other changes suggested by parents including making the P-MYMOP diabetes specific and changing the word “problem” to a simpler alternative were considered parent-centred and therefore were not incorporated into the adaptation of P-MYMOP.

#### Theme 6 Connecting activity limitation with identified problems

The third question on the P-MYMOP asked respondents to identify an activity limitation due to their identified problem(s).

Of the 24 included children, five left this column blank. Two children specifically wrote that there was no activity limitation. Three children were unable to understand the connection between activity limitation and identified problems.

One parent said that the activity question was completed by the child without considering the problems that the child mentioned. Another parent advised a change to the activity question from ‘now look at the faces below and circle the face that shows how hard it has been to do over the last few days’, to ‘now look at the faces below and circle the face that shows how hard it has been for you not to be able to do this.’ Based on this, the wording of the activity question was changed accordingly. Overall, children were able to connect the activity question to their identified problems. In cases where the mentioned activity was separated from the identified problems, the research team concluded that the activity question still provided useful information for the clinical consultation.

## Discussion

In this study the MYMOP [10], was adapted for children aged 7–11 years old. The results provide early evidence of content validation of the P-MYMOP for this population. The content validation was achieved by simplifying the tool’s wording, layout, and implementing a scaling method through four iterative stages. Children as young as 7 years old were able to understand and select faces to rate the severity of their symptoms without any help from parents. Inclusion of parent’s/guardian’s and children’s views in the adaptation of MYMOP is in line with current recommendations to consult the target population for the development of measurement tools.

Based on direct observation of the children’s completion of the P-MYMOP and interview responses, most children understood the concept of problems, the P-MYMOP’s recall period, and the faces scale. The medication questions were completed by the parents/guardians without any difficulty.

HRQOL is a subjective construct, so assessment can vary according to respondents. Parents/caregivers are often asked to provide an assessment of paediatric health outcomes, including measurement of subjective symptoms such as pain, anxiety, and depression. When children are non-verbal or pre-verbal, parents, caregivers, or healthcare providers may be an acceptable proxy for deriving paediatric outcomes. However, self-assessment of HRQOL should be encouraged where possible, given evidence that proxy ratings are systematically different from the child’s self-rated HRQOL [22–25]. Considerable discordance between children’s and parents’ ratings of HRQOL, psychological functioning, and physical functioning have been reported in children with chronic diseases [22–25]. The P-MYMOP is simple and designed to support self-rating of HRQOL in children aged 7–11 years old.

Valid self-completion of individualised HRQOL instruments requires respondents to identify their issues and rate them on a scale. With the adapted wording and layout of the P-MYMOP, most children were able to name their own problems, with occasional help from parents/guardians. The participating children confirmed in the interview that they understood all the questions on the P-MYMOP. The final adapted paediatric version of MYMOP is available from corresponding author on request.

To promote self-completion of the P-MYMOP by children, a faces scale was incorporated. Comparative use of scaling methods such as the ‘visual analogue scale’ (VAS), the ‘numeric rating scale,’ and the ‘faces scale’ have been studied in relation to pain measurement in children [25–27]. The use of faces is shown to improve self-reporting [27] and can be successfully understood by children as young as 4 years old [27]. The change from numeric rating scale to the ‘faces scale’ was further supported by the data collected during the child-parent interviews, and observation of the capability of children as young as 7 years old to understand and appropriately respond to the ‘faces scale’. When calculating the score of the P-MYMOP, the intention is to convert the measurements back to the 0–6 numeric-scale of the MYMOP. Similarly, the profile score is calculated by adding the score of each response and interpreting it parallel to the individual item scores. A qualitative comparison of sequentially completed MYMOP would be helpful in assessing any problem and show that the issue has improved/deteriorated overtime.

In the first iteration all respondents had suggested to change the word *symptom* to *problem.* However, the research team had decided to change it to “health problems,” to keep the MYMOP health centred. There was no change suggested to that wording in the second and third iterations. Before the fourth iteration, the P-MYMOP was piloted to two children (convenience sample — researcher’s own children) and based on their use and feedback, the wording was changed back to *problem* to keep the new measure open to any issues that the children may have. This was in line with keeping the P-MYMOP an individualised measure, with the realisation that children 7 to 11 years may have issues that may not be directly considered relevant to their health, but they may still be important for children and require support. The results of the fourth iteration of the study also support this.

In areas of paediatric research [28], the development of a generic individualised HRQOL measure for children consistently lags the development of adult measures. The MYMOP was developed in 1996 [29] and has been adapted for adult cancer patients [30–34], mental health outcomes [35–39], and elderly patients undergoing acupuncture [40, 41]. The MYMOP offers a simple and patient-centred approach for the measurement of HRQOL. The adaptation of this tool for children might enhance their clinical care by using a self-assessment of paediatric HRQOL which integrates their values and preferences.

Development of the P-MYMOP is an essential precursor to the first generic individualised assessment of paediatric HRQOL. Validity and reliability (or sound psychometric properties) are relative terms, and an instrument is only valid for its target population [18, 19]. Accordingly, development of tools for disease-specific populations and age-specific groups is challenging. Alternatively, generic individualised questionnaires can provide measurement of patient-specified domains or symptoms and is encouraged by the recent establishment of the Patient-reported Outcomes Information System (PROMIS) [39]. As stated by PROMIS researchers, domain-specific measures are the way forward, since the presence of a particular disease alone is not likely to define the experience of side-effects like fatigue, headache, sleep difficulty, anger, sadness, etc. [42]. In the current study, P-MYMOP was tested for the suitability of its content with children with a variety of health conditions. Despite differences in parent-reported diagnoses, children were able to complete the questionnaire successfully, and provide positive feedback when interviewed.

As with any measurement approach to HRQOL, there are some limitations when using individualised instruments for this purpose. Scores on individualised measures represent measurement of unique patient issues, and therefore cannot be used to discriminate between individual patients or patient groups, or for economic evaluations [1–4, 43, 44]. Furthermore, since the patients nominate their most important individualised domains/symptoms, changes in rank of importance over time may limit the evaluative properties of these tools longitudinally [1–4, 43, 44]. Some may argue that this would limit measurement properties of individualised instruments [45]. However, there are reasons to suggest that this does not affect their validity in clinical practice. For example, changes in nominated symptoms for evaluation can inform clinicians of the change in the patient’s experience and priorities over time thereby allowing clinicians to focus on other important aspects of life for the patient. Moreover, these limitations are shared by all individualised questionnaires and are not unique to the P-MYMOP. Individualised instruments can be beneficial in clinical consultations in primary, secondary, and tertiary-care settings, N-of-1 trials, and in randomised controlled trials to better understand heterogeneity of the treatment effect and derive individual level recommendations [46]. Importantly, individualised HRQOL measures can help patient and healthcare providers tailor healthcare according to the patient’s needs, values, and preferences.

No responses from paediatricians to the second online survey might be seen as a limitation of the study. However, the opinions of this group of users were collected via the first two iterations of the study. According to the guidance on validation of PROMs it is not necessary to sample a large group of service providers for validation [16, 47].

The P-MYMOP was developed through a top-down approach where the initial adult questionnaire underwent several rounds of adaptation through expert consultations followed by consultation with child-parent pairs [48, 49]. There are limitations to this approach especially when applied to tools with predetermined dimensions/domains, as adoption of a top-down method can mean that dimensions pertinent to a target population might be missed. The P-MYMOP however has no predetermined dimensions/domains. Each respondent (child) is given an opportunity to identify the domains/areas of life that are important to them and an opportunity to quantify their impact using the faces scale. During the adaptation, child-parent/child-guardian pairs were given opportunities to provide feedback on the wording, layout, response options, and recall period of the tool. However, additional interviews and/or focus groups with parents and children were undertaken to explore the feasibility of the study. While this may be crucial for the effective adaptation of standard PROMs, it is less helpful for individualised PROMs.

Integrating HRQOL assessment into routine clinical care can be challenging. Short, straightforward and quick individualised measures may help integrate HRQOL assessment in the time-constrained healthcare system. P-MYMOP is the first paediatric generic individualised HRQOL measure that offers a set of brief and easy questions which can be used to assess variation in patient-concerns regardless of their diagnosis. The P-MYMOP may also provide a useful source of information to assist in the understanding of inevitable heterogeneity of treatment effects. Given the global initiatives advocating for patient-centred research and outcomes [39, 50–53], and a better understanding that effectiveness evidence developed from clinical trials have limited application to individuals [53], P-MYMOP can provide more comprehensive data from the paediatric patients’ perspective. Individualised outcome assessment tools such as P-MYMOP hold much promise, given the rise in development of personalised medicine approaches aimed at tailoring conventional therapies from a patient-perspective.

## Conclusions

The P-MYMOP is the first generic individualised HRQOL measure created for children. The tool can be a starting point for individualised measurement of paediatric HRQOL. The wording, layout, and scale of the P-MYMOP has been successfully adapted for children aged 7–11 years old. Preliminary evidence on content validity has been generated, but as validation is an iterative process, further research is required to assess its feasibility, reliability, and construct validity.

## Data Availability

Data is available from the corresponding author upon reasonable request.

## Abbreviations

Patient/client/respondent: Used interchangeably
HRQOL: Health-related quality of life
ISOQOL: International Society of Quality of Life Research
MYMOP: Measure Yourself Medical Outcome Profile
P-MYMOP: Paediatric MYMOP
WCH: Women’s and Children’s Hospital
